# Anti-inflammatory effects of the prostaglandin D_2_/prostaglandin DP1 receptor and lipocalin-type prostaglandin D_2_ synthase/prostaglandin D_2_ pathways in bacteria-induced bovine endometrial tissue

**DOI:** 10.1186/s13567-022-01100-6

**Published:** 2022-11-26

**Authors:** Jindi Wu, Fan Bai, Wei Mao, Bo Liu, Xiaolin Yang, Jing Zhang, Tingting Li, Gerelt Borjigin, Jinshan Cao

**Affiliations:** 1 College of Food Science and Engineering, Inner Mongolia Agricultural University, Hohhot, 010018 China; 2 Laboratory of Veterinary Pharmacology, College of Veterinary Medicine, Inner Mongolia Agricultural University, Hohhot, 010018 China; 3 Key Laboratory of Clinical Diagnosis and Treatment Techniques for Animal Disease, Ministry of Agriculture, Hohhot, 010018 China; 4 Veterinary Research Institute, Inner Mongolia Academy of Agricultural and Animal Husbandry Sciences, Hohhot, 010031 China

**Keywords:** DP1 receptor, *Escherichia coli*, *Staphylococcus aureus*, anti-inflammatory effects, bovine endometrial tissue, L-PGDS/PGD_2_ pathway

## Abstract

Dairy cows often develop different degrees of endometritis after calving and this is attributed to pathogenic bacterial infections such as by *Escherichia coli* and *Staphylococcus aureus*. Infection of the bovine endometrium causes tissue damage and increases the expression of prostaglandin D_2_ (PGD_2_), which exerts anti-inflammatory effects on lung inflammation. However, the roles of PGD_2_ and its DP1 receptor in endometritis in cows remain unclear. Here, we examined the anti-inflammatory roles of the lipocalin-type prostaglandin D_2_ synthase (L-PGDS)/PGD_2_ and DP1 receptor regulatory pathways in bovine endometritis. We evaluated the regulatory effects of PGD_2_ on inflammation and tissue damage in *E. coli*- and *S. aureus*-infected bovine endometrial cells cultured in vitro. We found that the secretion of pro-inflammatory cytokines interleukin (IL)-6, IL-1β, and tumour necrosis factor (TNF)-α as well as expression of matrix metalloproteinase (MMP)-2, platelet-activating factor receptor (PAFR), and high mobility group box (HMGB)-1 were suppressed after DP1 receptor agonist treatment. In contrast, IL-6, IL-1β, and TNF-α release and MMP-2, PAFR, and HMGB-1 expression levels were increased after treatment of bovine endometrial tissue with DP1 receptor antagonists. DP1-induced anti-inflammatory effects were dependent on cellular signal transduction. The L-PGDS/PGD_2_ pathway and DP1 receptor induced anti-inflammatory effects in bovine endometrium infected with *S. aureus* and *E. coli* by inhibiting the mitogen-activated protein kinase and nuclear factor-κB signalling pathways, thereby reducing tissue damage. Overall, our findings provide important insights into the pathophysiological roles of PGD_2_ in bovine endometritis and establish a theoretical basis for applying prostaglandins or non-steroidal anti-inflammatory drugs for treating endometrial inflammatory infertility in bovines.

## Introduction

Bovine subfertility is the main cause of decreased fertility in cows [1]. Moreover, cows are prone to different degrees of endometritis after calving, most often because of infections with pathogenic bacteria, such as *Escherichia coli*. *Staphylococcus aureus* is another potential pathogen frequently isolated from the bovine uterine lumen and causes endometritis [2]. Such infections have great impact on fertilisation and conception if not treated appropriately and in a timely manner. The inflammatory response to bacterial infection in the cow uterus is designed to eliminate pathogens and repair damaged tissues [3]. In addition, prostaglandin D_2_ (PGD_2_) plays important roles in inhibiting the development of pulmonary inflammation and endometrial inflammation through its specific receptors [4]. However, the mechanisms mediating PGD_2_ expression in bovine endometritis induced by *E. coli* and *S. aureus* infections remain unclear.

Some studies have suggested that PGD_2_ exerts anti-inflammatory effects via D-type prostanoids (DPs) [5]. More than half of the cows are affected with various reproductive diseases, including endometritis and metritis, after delivery. Therefore, repairing the endometrium in postpartum cows is essential for facilitating subsequent pregnancies [6]. Using gene microarray analysis, Wei et al. [7] demonstrated that the expression of lipocalin-type prostaglandin D synthase (L-PGDS), a tumour cell inhibitor, is significantly downregulated during the growth of human uterine leiomyoma. Interestingly, PGD_2_ synthase expression in endometrial tissues is decreased during clinical bovine endometritis, and stimulation with lipopolysaccharide in vitro causes downregulation of PGD_2_ synthase in endometrial epithelial cells and stromal cells [8]. Thus, PGD_2_ and its receptors play important roles in the maintenance and health of the mammalian uterus physiology; these roles may be contradictory to those of the inflammatory mediator PGE_2_.

PGD_2_ exerts its biological functions via two different receptors, DP receptor 1 and chemoattractant receptor homologous molecule expressed on Th2 cells (CRTH2, also known as DP2), which are co-expressed on the eosinophil surface [9]. A deficiency of DP1 promotes vascular permeability, angiogenesis, and inflammatory cell infiltration in tumour growth in DP-deficient mice [10]. Moreover, DP1 agonists inhibit inflammation, angiogenesis, and tumour growth, suggesting the PGD_2_/DP1 signalling pathway as a new therapeutic target in cancer [11]. Recent studies have also confirmed that the PGD_2_/DP1 pathway can alleviate brain inflammation induced by infection with neurotropic coronavirus by increasing the expression of interferon-1 and pyrin domain-containing protein 3 (an anti-inflammatory protein) in microglial cells [12]. Thus, the PGD_2_/DP1 pathway is essential for mediating inflammatory processes in non-blood-derived cells [13].

Accordingly, in this study, we evaluated if the PGD_2_/DP1 pathway modulated inflammation and pathogen clearance by inhibiting the expression of inflammatory factors and chemokines to reduce damage to infected tissues in bovine endometritis. Our results provide insights useful for the treatment of bovine bacterial endometritis.

## Materials and methods

### Chemicals, reagents, and antibodies

The following chemicals, reagents, and other materials were used in this study: foetal bovine serum (ExCellBiology, Inc., Shanghai, China); Dulbecco’s modified Eagle medium (DMEM)/F-12, penicillin, and streptomycin (Gibco, Grand Island, NY, USA); amphotericin B (GENERAY, Shanghai, China); bovine interleukin (IL)-6 enzyme-linked immunosorbent assay (ELISA) reagent kit (DY8190) and bovine tumour necrosis factor (TNF)-α duo set (DY2279; R&D Systems, Minneapolis, MN, USA); bovine IL-1β ELISA reagent kit (ESS0027; Kingfisher Biotech, St. Paul, MN, USA); Six-well culture plates (Corning, Inc., Corning, NY, USA); T-PER tissue protein extraction reagent, Halt Protease Inhibitor, Pierce BCA Protein Assay Kit, and prestained protein ladder (Thermo Fisher Scientific, Waltham, MA, USA); sodium dodecyl sulphate polyacrylamide gel electrophoresis (SDS-PAGE) loading buffer (TAKARA, Shiga, Japan); centrifugal filter units (Millipore, Billerica, MA, USA); SDS-PAGE kit (Solarbio, Beijing, China); 10X Tris/Glycine buffer (Bio-Rad Laboratories, Hercules, CA, USA); transfer membranes (Millipore); Starting Block T20 (TBS) Blocking Buffer (Thermo Fisher Scientific); Halt Protease Inhibitor (Thermo Fisher Scientific); antibody dilution reagent (Beyotime, Shanghai, China); anti-matrix metalloproteinase (MMP)-2 antibody (Abcam, ab97779, Cambridge, UK); anti-platelet-activating factor receptor (PAFR) antibody (Biorbyt, orb11225, Cambridge, UK); and anti-high mobility group box (HMGB)-1 antibody (Novus Bio, NB100-2322, Littleton, CO, USA); Goat anti-rabbit IgG horseradish peroxidase-linked antibodies and goat anti-mouse IgG horseradish peroxidase-linked antibodies (Cell Signaling Technology, 7074 and 7076, Danvers, MA, USA); goat anti-rabbit IgG H&L antibodies (Alexa Fluor 647) preadsorbed (Abcam, ab150083, Cambridge, UK); AxyPrep Multisource Total mRNA Miniprep Kit (Axygen Scientific, Union City, CA, USA); Primer Script RT Master Mix (Takara); FastStart Universal SYBR Green Master (Rox; Roche, Basel, Switzerland); Luria Bertani broth (Oxoid, Hampshire, UK); Mueller-Hinton II cation adjusted broth (MH broth; BD Biosciences, Franklin Lakes, NJ, USA); and optimal cutting temperature compound (Sakura, Torrance, CA, USA). All primers were synthesised by Invitrogen (Carlsbad, CA, USA). All inhibitors used in this study are listed in Table 1.

**Table 1:** **Agonists, antagonists, and inhibitors used in this study**

Reagent	Function	Company
BW-245 C	DP1 agonist	Cayman
15d-PGJ_2_	DP1 agonist	Cayman
S5751	DP1 antagonist	Cayman
MK-0524	DP1 antagonist	Cayman
AT56	L-PGDS inhibitor	Cayman
CAY10678	L-PGDS inhibitor	Cayman

### Collection and cultivation of endometrial tissue in vitro

The uterine horn was collected from 50 cows (age: 24 months; weight: approximately 600 kg) with no evident genital disease or microbial infection. All tissues were kept in chilled sterile phosphate-buffered saline (PBS) and transported to our laboratory until further processing within 1 h. Briefly, the tissues were washed three times with PBS supplemented with 100 U/mL penicillin and streptomycin and 2.5 µg/mL amphotericin B and then incubated at 4 °C for 1 h. Next, all tissues were excised under aseptic conditions and opened longitudinally. Endometrial tissues containing epithelial and stromal cells were removed from the endometrial region using curved scissors and ophthalmic tweezers under aseptic conditions, and the tissues were subdivided into pieces measuring approximately 2 mm in diameter and 1 mm in thickness. The explants were placed randomly in 6-well plates pre-coated with 3 mg/mL rat tail collagen and supplemented with 3.75 mL of the culture medium (DMEM/F-12) supplemented with 20% foetal bovine serum, 100 U/mL penicillin and streptomycin, and 2.5 µg/mL amphotericin B [14]. Endometrial explants were incubated in a humidified and sterile environment (5% CO_2_, 37 °C) for 24 h before the next treatment. One assay was performed using tissues isolated from the same uterine horn.

### Preparation of bacterial suspensions

Pathogenic *E. coli*-infected endometrial tissues were isolated from the uteri of bovines with clinical endometritis (Identification Certificate Number SYS110017). *Staphylococcus aureus* was isolated from the uteri of bovines with clinical endometritis (Identification Certificate Number SYS110018). Bacteria were cultured in the Luria Bertani broth and Mueller-Hinton II cation adjusted broth at 37 °C for 16 h with constant shaking to an optical density of 2.0 at 600 nm. The cultures were centrifuged at 5000 × *g* for 6 min at 4 °C and then resuspended with sterile PBS. The above-mentioned step was repeated three times, and the cells were finally resuspended in tissue culture medium.

### Experimental treatments

Bovine endometrial tissue fragments were washed three times with PBS prior to bacterial stimulation. Next, 3.5 mL of DMEM/F-12 containing 20% foetal bovine serum was added to each well. Endometrial tissue fragments were treated as follows: control, bovine tissue cultured under normal physiological conditions; pathogenic group, *E. coli* and *S. aureus*; *E. coli + S. aureus +* DP1 antagonist groups; *E. coli + S. aureus +* DP1 agonist groups; and *E. coli + S. aureus +* L-PGD inhibitor groups. Live endometrial pathogenic *E. coli* and *S. aureus* cells (1 × 10^6^ colony forming units/mL) were added to all non-control wells. DP1 agonists were used at a concentration of 10^− 5^ M. DP1 antagonists and L-PGD inhibitors were used at a concentration of 10^− 6^ M, as determined in preliminary experiments.

### Real-time reverse transcription polymerase chain reaction (qPCR) analysis

Total mRNA extraction, reverse transcription, and qPCR were conducted to measure gene expression. Total cDNA was used as a template for qPCR with the FastStart Universal SYBR Green Master on an iCycleriQ5 real-time PCR detection system (Bio-Rad). PCR conditions were as follows: 50 °C for 2 min, 95 °C for 10 min, followed by 40 cycles of amplification at 95 °C for 15 s and 60 °C for 60 s. *β-Actin* was used as a reference gene. The expression and activity of β-actin were stabilised in cells. Differences in gene expression were calculated using the 2^−△△Ct^ method. All primers used for qPCR are listed in Table 2.

**Table 2:** **Primer sequences for qPCR**

Gene name	Accession No.	Sequence (5′–3′)	Tm (°C)	Concentration (nM)
*β-actin*	NM_173979.3	F:CCAAGGCCAACCGTGAGAAGAT	54	400
		R:CCACGTTCCGTGAGGATCTTCA		
*MMP-2*	NM_174745.2	F:GGCATCTCTCAGATCCGTGG	55	400
		R:TGTGGGTCTTCGTACACAGC		
*PAFR*	NM_001040538.1	F:TACTGCTCAGTGGCCTTCCT	55	400
		R:AATGTTCAAAGCAGCGTGTG		
*IL-6*	NM_173923.2	F: ATGCTTCCAATCTGGGTTC	52	400
		R:TGAGGATAATCTTTGCGTTC		
*TNF-α*	NM_173966.3	F:ACGGGCTTTACCTCATCTACTC	56	400
		R:GCTCTTGATGGCAGACAGG		
*IL-1β*	NM_174093.1	F:AGGTGGTGTCGGTCATCGT	56	400
		R:GCTCTCTGTCCTGGAGTTTGC		

### ELISA

The tissues were subdivided into pieces measuring approximately 2 mm in diameter and 1 mm in thickness, and were randomly and equally distributed to each well. Supernatants of tissues cultured in 6-well plates were centrifuged at 300 × *g* for 8 min at 4 °C and then stored at −80 °C. The concentrations of IL-1β, IL-6, and TNF-α (in 100 µL) were measured using ELISA kits according to the manufacturer’s instructions. Three biological replicates were performed.

### Western blot analysis

For Western blotting analysis, 25 µg of the total protein was resolved by SDS-PAGE in each lane of an SDS polyacrylamide gel and blotted onto polyvinylidene difluoride membranes. The membranes were blocked with Starting Block (TBS) Blocking Buffer at 4 °C and then incubated with primary antibodies for 16 h at 4 °C. Rabbit anti-phospho-extracellular signal-regulated kinase (ERK), anti-ERK, anti-phospho-p38, anti-p38, anti-phospho-nuclear factor (NF)-κBp65, and anti-NF-κBp65 monoclonal antibodies (1:1000) as well as anti-β-actin antibodies (1:1000) were used for protein detection [15]. The inhibitor-specific dilutions were 1:100 for PAFR, 1:100 for MMP-2, and 1:100 for HMGB-1 [16]. Proteins were visualised using secondary horseradish peroxidase-conjugated goat anti-rabbit or goat anti-mouse antibodies (1:3000) and Pierce SuperSignal West Femto chemiluminescent substrate. Grey-scale values of bands generated by Western blotting were measured using ImageJ software (National Institutes of Health, Bethesda, MD, USA). The band density of the target proteins was normalised to that of the β-actin band obtained for the same samples.

### Double-labelling immunofluorescence assays

Frozen Sect. (6 μm) of treated endometrial explants were thawed at room temperature for 15 min and fixed in cold acetone for 10 min. The thawed sections were blocked for 1 h in 3% bovine serum albumin at 25 °C. The primary antibodies (anti-MMP-2 [1:100], anti-HMGB-1 [1:100]) were added, and the sections were incubated overnight at 4 °C in the dark [16]. Following incubation, the slides were incubated in a 1:1000 dilution of secondary donkey anti-rabbit IgG H&L antibodies (Alexa Fluor 647) for 1 h at room temperature (25 °C ± 1 °C). Confocal microscopy (LSM 800; Zeiss, Oberkochen, Germany) was used to capture images (400× magnification) and analyse the fluorescence intensity.

### Immunohistochemical staining

Treated bovine endometrial tissues were prepared into paraffin sections (10 μm). The sections were then dehydrated through a gradient of alcohol concentrations, and endogenous enzyme was inactivated using 3% H_2_O_2_. Next, the slices were immersed in 95 °C citric acid hydrochloride buffer for 30 min. The sections were blocked with 5% bovine serum albumin at room temperature (25 °C ± 1 °C) for 1 h. The sections were then incubated with primary antibodies (anti-MMP-2 [1:100], anti-PAFR [1:100], anti-HMGB-1 [1:100]) without washing overnight at 4 °C in the dark [16]. After incubation, the slides were incubated in a 1:1000 dilution of the secondary antibody for 1 h at 37 °C and analysed using a Liquid DAB + Substrate Chromogen System, according to the manufacturer’s instructions. Finally, the sections were counterstained with haematoxylin for 5 min and decolourised through a gradient of alcohol concentrations. An optical microscope was used to capture images and analyse fluorescence intensity.

### Data analysis

All data were analysed using GraphPad Prism 6 (GraphPad, Inc., San Diego, CA, USA) and are presented as the means ± standard deviations. Statistical significance was evaluated by one-way analysis of variance (ANOVA), followed by a post-hoc analysis (Dunnett’s test) to control for the number of comparisons (*n* = 5). Results with *P* values ≤ 0.05 were considered as statistically significant. The ImageJ and GraphPad Prism 5 software were used to visualise the results.

## Results

### Agonists and/or antagonists of DP1 and inhibitors of L-PGDS regulate PAFR and MMP-2 expression in *E. coli*- and *S. aureus*-challenged ex vivo endometrial explants

**Figure 1:**
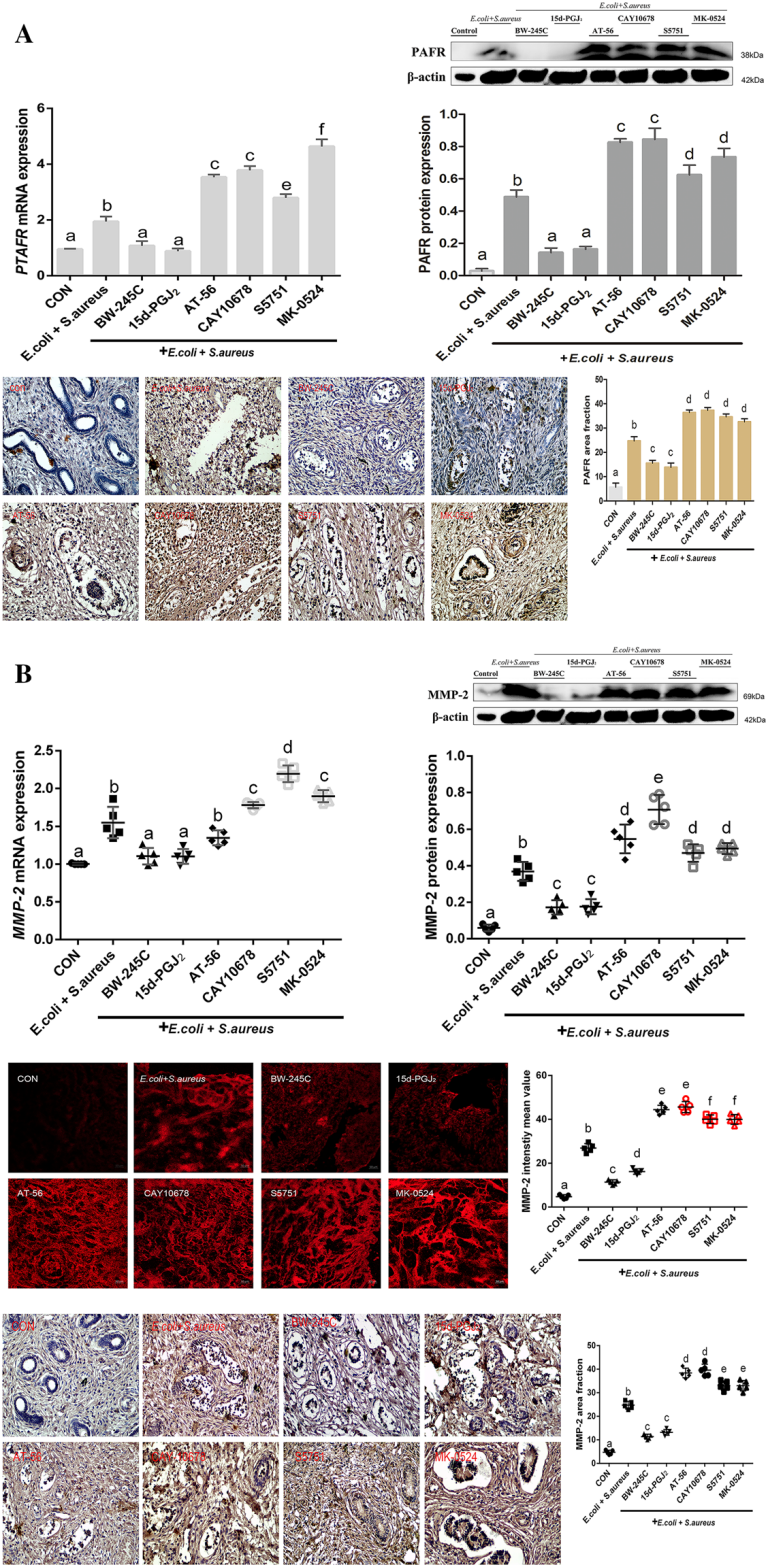
**PGD**_**2**_**-mediated regulation of PAFR and MMP-2 via the L-PGDS/PGD**_**2**_**and PGD**_**2**_**/DP1 pathways in** ***E. coli*- and** ***S. aureus*-infected bovine endometrial explants.** Immunofluorescence staining, Western blotting, and qPCR results for PAFR (**A**) and MMP-2 (**B**). Data are given as the means ± SEMs. The significance of differences between results was determined by one-way ANOVA, followed by the Dunnett’s test to control for the number of comparisons (*n* = 3). Different letters indicate significantly different means (*P* < 0.05).

First, we explored the associations among PGD_2_/DP1, pro-inflammatory cytokine (PAFR), and wound healing-related molecules (MMP-2) in bovine endometrial explants infected with *E. coli* and *S. aureus* by qPCR and Western blotting. The mRNA and protein expression levels of PAFR and MMP-2 were significantly higher in the *E. coli*- and *S. aureus*-infected groups than in the control group (*P* < 0.05) (Figure 1). However, treatment with the DP1 agonists BW-245 C and 15d-PGJ_2_ markedly reduced the expression of PAFR and MMP-2 in the *E. coli-* and *S. aureus*-infected groups (*P* < 0.05). Immunofluorescence analysis indicated that the fluorescent intensity of MMP-2 (Figure 1B) in the explants was consistent with the Western blotting results (*P* < 0.05). The immunohistochemistry results for PAFR (Figure 1A) and MMP-2 (Figure 1B) were also similar to the Western blotting results (*P* < 0.05). In addition, treatment with the DP1 inhibitors S5751 and MK-0524 and L-PGDs inhibitors AT-56 and CAY10678 increased the expression of PAFR (Figure 1A) and MMP-2 (Figure 1B) during infection (*P* < 0.05). These results indicate that the PGD_2_/DP1 pathway plays an anti-inflammatory role mediated by L-PGDS during *E. coli* and *S. aureus* infection by inducing PAFR and MMP-2 expression in bovine endometrial explants in vitro.

### Agonists and/or antagonists of DP1 and inhibitors of L-PGDS regulate the release of inflammatory factors via the MAPK and NF-κB signalling pathways in bacteria-challenged ex vivo endometrial explants

**Figure 2:**
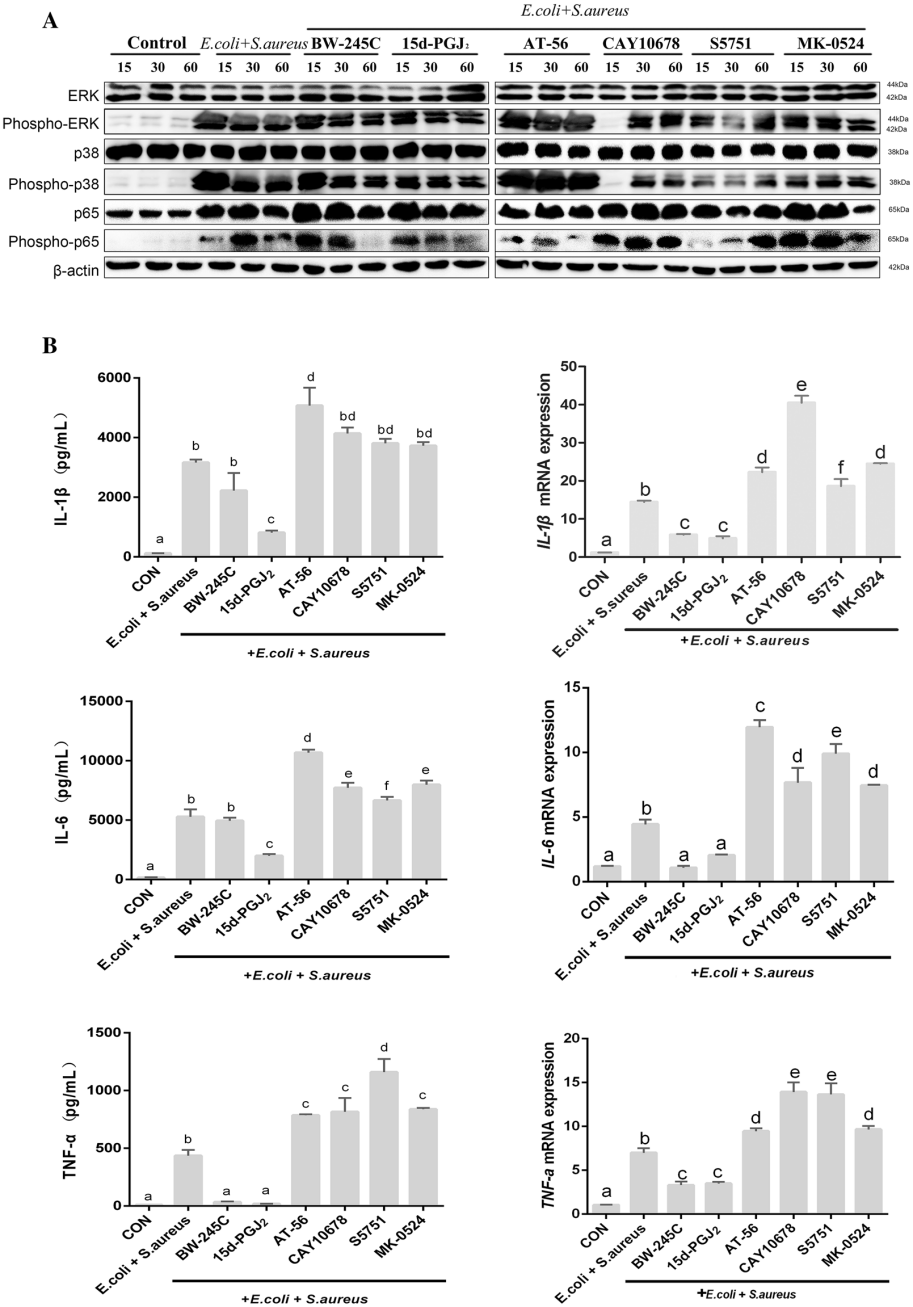
**L-PGDS/PGD**_**2**_ **and PGD**_**2**_**/DP1 mediated IL-6, IL-1β, and TNF-α secretion via the ERK/NF-κB and p38/NF-κB signalling pathways in** ***E. coli*- and** ***S. aureus*-infected bovine endometrial explants.** Phosphorylation of ERK1/2, p38, and p65 was detected by Western blotting analysis (**A**). qPCR and ELISA results for IL-6, IL-1β, and TNF-α (**B**).

To investigate if PGD_2_ was associated with the production of pro-inflammatory cytokines in *E. coli*- and *S. aureus*-infected endometrial explants, we determined the expression of *IL-6*, *IL-1β*, and *TNF-α* by qPCR and secretion of IL-6, IL-1β, and TNF-α by ELISA. As shown in Figure 2, infection with both bacteria and DP1 antagonists significantly reduced the mRNA expression and secretion levels of IL-6, IL-1β, and TNF-α compared with those in the bacteria-only group (*P* < 0.05). However, the expression and secretion of IL-6, IL-1β, and TNF-α were significantly increased upon treatment with DP1 antagonists and L-PGDS inhibitors compared with those in the bacteria-only group (*P* < 0.05). To evaluate the effects of PGD_2_ on *E. coli*- and *S. aureus*-induced MAPK and NF-κB signalling in the bovine endometrium, activation of ERK, p38, and p65 was examined by Western blotting. In the MAPK pathway, DP1 antagonists impaired p38 and ERK phosphorylation relative to that in bacteria-infected explants (Figure 2A). Moreover, treatment with DP1 antagonists and L-PGDS inhibitors enhanced the activation of ERK, p38, and p65 compared with that in the bacteria-only group. Interestingly, activation of these proteins following treatment with DP1 antagonists was not as high as that of proteins treated with L-PGDS inhibitors. Additionally, *E. coli* and *S. aureus* significantly induced MAPK and NF-κB phosphorylation in the bovine endometrium at 15, 30, and 60 min after infection than in the uninfected control group (*P* < 0.05). These MAPK pathway activation data were consistent with the results of cytokine release (Figure 2A). Collectively, our data indicate that PGD_2_ is involved in anti-inflammatory processes through the MAPK/NF-κB pathway in the bacteria-infected endometrium.

### Morphology and HMGB-1 expression in bovine endometrial explants after bacterial infection

**Figure 3:**
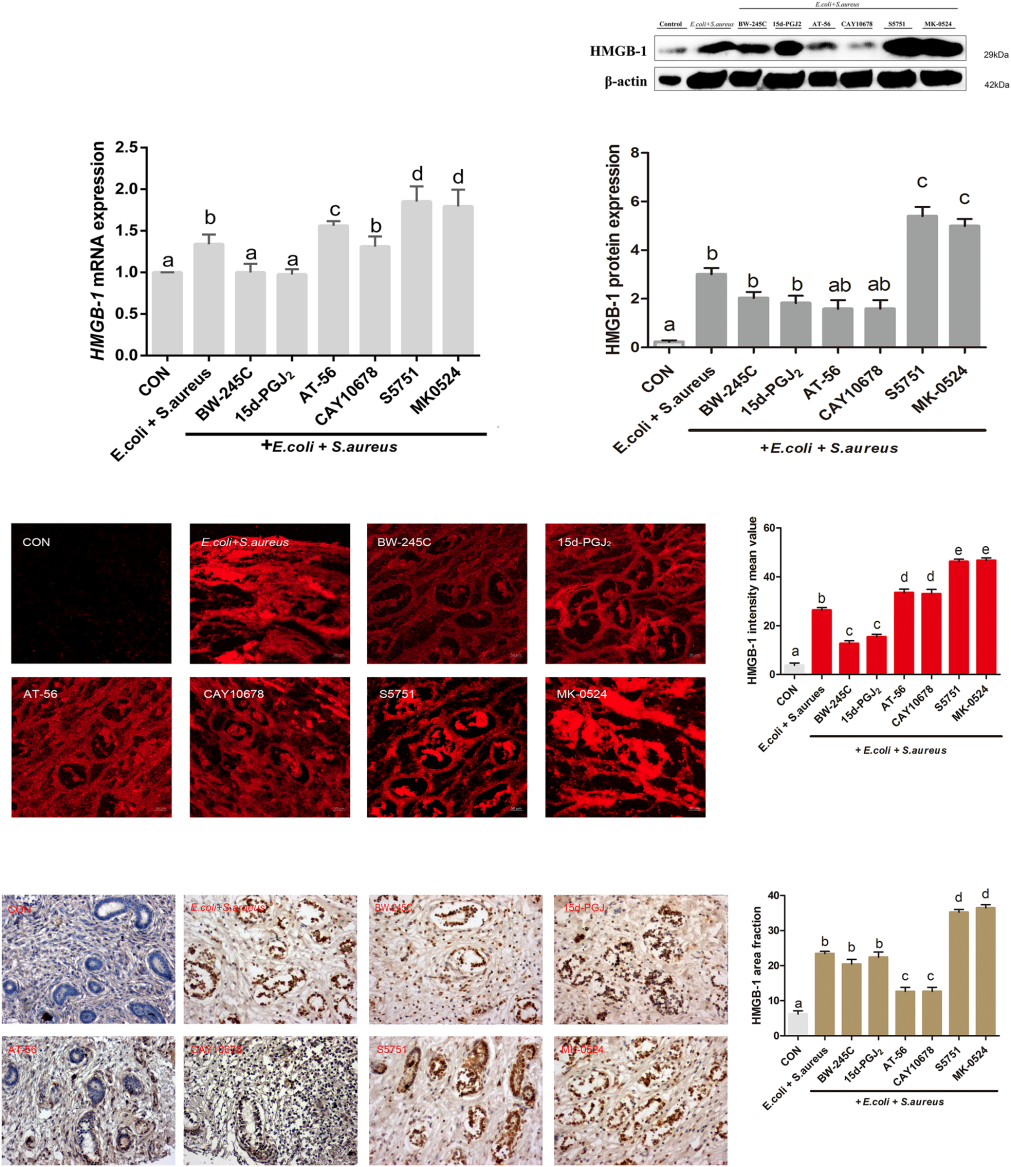
**PGD**_**2**_**-mediated regulation of HMGB-1 via L-PGDS/PGD**_**2**_ **and PGD**_**2**_**/DP1 pathways in** ***E. coli*- and** ***S. aureus*-infected bovine endometrial explants**. Immunofluorescence staining, immunohistochemical staining, Western blotting, and RT-PCR results for HMGB-1 are shown. Data are presented as the means ± SEMs. The significance of differences between results was determined by one-way ANOVA, followed by the Dunnett’s test to control for the number of comparisons (*n* = 3). Different letters indicate significantly different means (*P* < 0.05).

We observed that the PGD_2_/MAPK/NF-κB pathway was essential for the anti-inflammatory effects in bovine endometritis (Figure 2A). Therefore, the potential involvement of PGD_2_ in inducing tissue damage was evaluated. The expression of HMGB-1 was determined by Western blotting and immunofluorescence staining. As shown in Figure 3, the expression of HMGB-1 in bovine endometrial explants was reduced by PGD_2_ inhibitors after infection. The expression level of HMGB-1 was significantly lower in the L-PGDS inhibitor-treated group than in the bacteria-only group (*P* < 0.05). However, HMGB-1 expression in bovine endometrial explants was not influenced by DP1 receptor agonists or antagonists after infection. The immunofluorescence intensities of HMGB-1 were consistent with the protein levels determined by Western blotting. These findings indicate that PGD_2_ plays a protective role via the L-PGDS/PGD_2_ pathway but not the PGD_2_/DP1 pathway in tissue damage observed in bovine endometritis.

## Discussion

Reproductive tract inflammatory diseases represent a failure of the immune system to shift from the downregulated state necessary for maintenance of pregnancy to a heightened state of function for postpartum clearance of bacteria and tissue debris and back to low levels of inflammation 3–4 weeks later [17]. Bacterial infection with one or more recognised pathogens, such as *E. coli*, initiates inflammation of the uterus. *Staphylococcus aureus* has also been isolated from the uterus of cows with endometritis. LeBlanc [6] found that approximately 30–50% of the cows have subclinical inflammation of the uterus after delivery. Few management practices or interventions specifically prevent endometritis. In most cows, this anti-inflammatory process leads to clearance of bacterial infection and eventual repair of the epithelium, at which point inflammation is decreased. Therefore, the anti-inflammatory process and repair of the uterine membrane of cows after delivery play important roles in reconnection [6].

Recently, we have shown that endogenous PGE_2_ regulates *S. aureus*-induced cytokine secretion from macrophageswhich proved that PGE_2_ would had a certain pro-inflammatory effect. We found that TNF-α secretion was enhanced, while IL-1β secretion was decreased in S. aureus-infected macrophages when endogenous PGE2 production was blocked [18]. Because of the above studies, we were more eager to explore the role of PGD2 in the process of inflammatory response. Studies have shown that PGD_2_ has essential roles in various physiological processes, particularly in several steps of the reproductive cycle and L-PGDS are widely expressed in the male reproductive system [19]. Additionally, PGD_2_ together with the prostaglandin PGE_2_ are involved in the inflammation process. Stimulation of DP1 receptors are known to activate adenyl cyclase and increase intracellular cAMP levels and PKA activity, though DP1 receptor signaling has yet to be extensively characterized [20]. DP1 is widely expressed and is present in several types of haematological and non-haematological cells [21]. Although DP1 receptor antagonist(S5751) markedly inhibited allergic pulmonary inflammation in a guinea-pig model of asthma [22]. DP1-mediated signals suppress cell migration and/or activation of DP-transfected Jurkat cells, eosinophils, basophils, dendritic cells, and fibroblasts, suggesting that DP1 has an anti-inflammatory role in immune system cells [23, 24]. This study also proved that DP1 mainly plays an anti-inflammatory role in the process of endometritis in dairy cows. In addition, PGD_2_ is a key mediator of inflammatory diseases, where it is likely to have dual roles, both in promoting and inhibiting inflammatory processes depending on the animal species, organ, and inflammatory stimulus [25]. Therefore, how (L-PGDS)/PGD2 and DP1 receptors play an anti-inflammatory role in bacterial infective cow endometritis is of important research significance. However, the anti-inflammatory mechanism and protective effects of PGD_2_ in cow endometritis remain unknown. Previous studies showed that cyclooxygenase-2 and mPGES-1 inhibitors significantly decrease PAFR and MMP-2 expression in *E. coli*-challenged ex vivo endometrial explants via the expression and secretion of IL-1β, IL-6, and TNF-α [16]. In the present study, we showed that PGD2/DP1 may play a vital role in anti-inflammatory responses by inhibiting activation of MAPKs and the NF-κB signalling pathway in bovine bacteria-induced endometritis. Furthermore, another study showed that ERK and NF-κB inhibitors alleviated the inflammatory reaction by decreasing the production of PGE_2_, IL-6, and TNF-α, and HMGB-1 in bacteria-infected bovine endometrial tissues, thereby mediating tissue damage [26]. Interestingly, this study showed that the PGD_2_/DP1 pathway inhibited the phosphorylation of MAPK and NF-κB signalling pathway components during the inflammatory response.

PAFR plays an important role in leukocyte recruitment and tissue injury, it may accelerate collagenolysis by increasing local concentrations of IL-6 that induce migration of leukocytes into the cervix, contributing to the promotion of cervical ripening during parturition [27, 28]. In the current study, PAFR expression was strongly upregulated in endometrial tissue infected with *E. coli* and *S. aureus*, similar to a previous report demonstrating significantly increased PAFR expression in human monocyte-like cells [29]. MMP-2 activity promotes angiogenesis and neovascularisation that the role of MMP-2 through PGE2-mediated pathway for the promotion of angiogenesis in endometriosis [30, 31]. Additionally, our in vivo studies demonstrated that DP1 agonists effectively inhibited the increased expression of PAFR and MMP-2 in these explants, consistent with the above findings. We showed that S5751, MK-0524, AT-56, and CAY10678 promoted PAFR and MMP-2 production in *E. coli*- and *S. aureus*-challenged ex vivo endometrial explants by targeting DP1 and L-PGDS, the essential receptor and enzyme regulating anti-inflammatory PGD_2_ synthesis. Furthermore, DP1 may be involved in this process.

Notably, downregulation of HMGB1 in inflammation ameliorates innate immune responses [32]. Additionally, HMGB1 is a typical intracellular tissue damage-related factor released in necrotic or damaged cells and promotes inflammatory injury. The combination of HMGB1 and IL-1β induces the expression of IL-6 [32]. To specifically clarify the damage to endometrial tissue, we examined the expression of HMGB1. Our results showed that HMGB-1 expression was not similar to that of the other three inflammatory factors examined above. Indeed, DP1 receptor agonists inhibited the expression of HMGB1, whereas DP1 antagonists promoted HMGB1 expression. Nevertheless, inhibitors of L-PGD did not promote HMGB1 expression. These findings suggest that the PGD_2_/DP1 pathway regulates HMGB1, whereas the L-PGDS/PGD_2_ pathway may not participate in this process. HMGB1 was also upregulated after treatment with DP1 antagonists, consistent with the further deterioration of endometrial tissue. Thus, the PGD_2_/DP1 pathway may protect against tissue damage in bovine endometritis.

The anti-inflammatory effects of PGD2/DP1 and L-PGDS/PGD2 pathways during the process of endometritis in dairy cows are mainly reflected in two aspects. First, we demonstrated that PGD_2_ inhibited the secretion of the inflammatory cytokines IL-1β, IL-6, and TNF-α to some extent, contributing to inhibition of the phosphorylation of p38, ERK, and NF-κB in bovine endometritis. Second, the PGD_2_/DP1 pathway significantly may have protective effects on tissue damage in bovine endometritis. Based on these findings, BW-245 C and 15d-PGJ_2_ may act as endogenous suppressors of inflammatory diseases, including bovine endometritis. Improving our understanding of the mechanisms involved in bovine endometritis may reveal novel targets for the development of anti-inflammatory drugs.
